# Editorial: Exploring the role of exercise in hypertension and blood pressure variability

**DOI:** 10.3389/fcvm.2026.1836245

**Published:** 2026-04-10

**Authors:** Giuseppe Caminiti, Marco Alfonso Perrone

**Affiliations:** 1 Department of Human Science and Promotion of Quality of Life, San Raffaele Open University, Rome, Italy; 2 Cardiology Rehabilitation Unit, IRCCS San Raffaele, Rome, Italy; 3 Division of Cardiology and Sports Medicine, Department of Clinical Sciences and Translational Medicine, University of Rome Tor Vergata, Rome, Italy

**Keywords:** blood pressure variability, cardiovascular prevention, exercise training, hypertension, lifestyle habits

Arterial hypertension (AH) remains a leading modifiable cardiovascular risk factor, affecting over 1.3 billion adults worldwide and substantially contributing to the global burden of cardiovascular and cerebrovascular diseases, thus representing a major public health challenge (1). Beyond mean BP levels, BP variability (BPV), the oscillation of BP over time, has also emerged as an independent predictor of cardiovascular events (2). The management of AH and BPV requires a multifaceted approach, encompassing both long-term pharmacological therapy and the adoption of healthy lifestyle habits. Among lifestyle strategies, exercise training (ET) is widely recognized as an effective intervention for preventing increases in BP and reducing BP levels in individuals with established AH. Nevertheless, several aspects of exercise prescription for BP control remain insufficiently defined. Moreover, evidence on the effectiveness of ET in reducing BPV remains scarce and largely anecdotal. Manuscripts included in this Research Topic address: a) comparisons of the antihypertensive effectiveness of ET programs; b) the relationship between exercise tolerance and AH; c) the role of ET in counteracting some key pathophysiological mechanisms underlying increases in BP and BPV. Although numerous studies have demonstrated the efficacy of ET in lowering BP, relatively few have directly compared the BP-lowering effects of different exercise modalities. In this context, the meta-analyses by Liu et al. and Zhang et al. provide consistent and complementary evidence. Liu et al., analyzing 68 randomized controlled trials (RCTs), compared multiple exercise modalities and found that light-intensity continuous training combined with antihypertensive medication was the most effective intervention for reducing systolic BP, whereas the traditional Chinese exercise Wuqinxi combined with medication achieved the greatest reduction in diastolic BP. Similarly, Zhang et al., including 29 RCTs focused on traditional Chinese exercises, reported that Tai Chi combined with antihypertensive therapy yielded the largest decrease in systolic BP, while Wuqinxi remained the most effective for diastolic BP reduction. Dance-based exercise has also shown beneficial effects on BP regulation, with previous studies reporting reductions of 12.0 mmHg in systolic BP and 3.4 mmHg in diastolic BP following structured dance therapy programs (3). However, substantial heterogeneity in exercise modality, training frequency, and program duration limits the identification of an optimal exercise “dose.” Addressing this gap, the meta-analysis by Wang et al., based on 11 RCTs, suggests that programs lasting ≥12 weeks, with sessions performed at least three times per week and up to 60 min in duration, are associated with greater BP reductions. Robust evidence supports an inverse relationship between cardiorespiratory fitness and the risk of developing hypertension (4, 5); however, further research is needed to better characterize this association in specific populations. Using data from American and Chinese registries, Tan et al. investigated the relationship between hypertension and non-exercise estimated cardiorespiratory fitness (NEE-CRF), demonstrating that each unit increase in NEE-CRF was associated with a lower risk of hypertension and reduced all-cause mortality in both cohorts. While consistent with previous findings, this study is notable for confirming similar associations across populations with marked differences in ethnicity, lifestyle, and environmental factors. In a cross-sectional study, Wu et al. examined the association between lifestyle behaviors, including physical activity, and hypertension across BMI categories. Notably, among underweight individuals, BP control appeared more favorable in those reporting lower levels of physical activity. This unexpected observation may reflect differences in basal metabolic rate or energy balance, as suggested by the authors, but warrants further investigation and validation in Western populations. In another elegant cross-sectional study in a Chinese cohort, Luo et al. assessed the relationship between AH and health-related physical fitness parameters, showing that individuals with AH exhibited impaired balance and reduced aerobic endurance compared with normotensive controls. These findings are clinically relevant, as identifying specific deficits in physical fitness may support the development of more individualized ET programs aimed at preventing BP elevation or mitigating AH. High maximal aerobic capacity, assessed by cardiopulmonary exercise testing, is associated with lower resting BP values and improved cardiovascular health in hypertensive individuals. However, differences exist between cardiopulmonary parameters obtained using treadmill vs. cycle ergometer testing, highlighting the need for standardized protocols and cross-center comparability, as emphasized by Leonardi et al. in a cohort of adult patients with congenital heart disease. Dysfunction of the autonomic nervous system (ANS) and increased arterial stiffness play key roles in the development and progression of AH and represent major targets of ET programs aimed at reducing BP (6, 7). The meta-analysis by Wang et al. evaluated the effects of different ET modalities on the sympathetic and parasympathetic branches of the ANS, demonstrating that combined (aerobic plus resistance) exercise had the greatest impact on systolic and diastolic BP, diastolic BPV, and sympathetic activity in hypertensive patients. Similarly, the meta-analysis by Xi et al. investigated the most effective exercise modalities for improving arterial stiffness in hypertensive and pre-hypertensive individuals. Their findings indicated that aerobic exercise, particularly when performed at moderate intensity, with a frequency of at least three sessions per week and a total weekly duration of ≥180 min, significantly improved arterial stiffness in these populations. Overall, these results contribute to defining an effective “exercise dose” capable of improving ANS and vascular function and reducing cardiovascular risk in individuals with AH. The implementation of effective interventions, including ET, to reduce the socio-health burden of AH is closely linked to public awareness of the condition (8). In the study by Chekol et al., less than half of hypertensive patients in Ethiopia demonstrated adequate knowledge of risk factors and complications, with social media use identified as a predictor of awareness. These findings underscore the need for comprehensive educational strategies alongside treatment, including the strategic use of social media. In conclusion, the findings of the original articles and meta-analyses of this Research Topic contribute to deepen our knowledge on the role of ET in the management of AH and BPV. At the same time, they move in the direction of a more individually tailored ET prescription. Nevertheless, further high-quality, large-scale studies are warranted to better define the optimal exercise prescription for BP and BPV control across diverse populations, as well as to elucidate underlying mechanisms, thereby supporting the development of future dedicated Research Topics in this evolving field.
